# A bacteria-based system expressing anti-TNF-α nanobody for enhanced cancer immunotherapy

**DOI:** 10.1038/s41392-023-01364-0

**Published:** 2023-04-19

**Authors:** Lina Liu, Xing Liu, Wenjie Xin, Lulu Zhou, Baolian Huang, Chao Han, Zhiting Cao, Zichun Hua

**Affiliations:** 1 The State Key Laboratory of Pharmaceutical Biotechnology, School of Life Sciences, Nanjing University, Nanjing, 210023 Jiangsu China; 2 School of Biopharmacy, China Pharmaceutical University, Nanjing, 210023 Jiangsu China; 3 Changzhou High-Tech Research Institute of Nanjing University and Jiangsu TargetPharma Laboratories Inc., Changzhou, 213164 Jiangsu China

**Keywords:** Drug delivery, Immunotherapy, Drug development

**Dear Editor**,

As a successful drug for inflammatory diseases, the application of TNF-α inhibitor on cancer therapy is limited by repeated administration and off-target effects.^1^ A body of evidence indicated that the anti-tumor efficacy of TNF-α inhibitor is unsatisfactory, though repeated administration was used to improve its efficacy in tumor-treating fields, it will also lead to severe side effects and high cost.^2–4^ Hence, an efficient and highly targeted TNF-α antibody delivery system is worth developing.

The genetically modified strain of attenuated *Salmonella typhimurium* VNP20009 (VNP) not only has super tumor-targeting capacity and genetic stability in vivo, but also has thousands of times higher enrichment in tumors than that of liver and spleen.^5^ Thus, in this work, we built a novel VNP delivery system expressing anti-TNF-α nanobody (VNP_αTNF-α_) (Fig. 1a and Supplementary Fig. S1a–g), which could significantly improve the delivery efficiency by continuous release of the nanobody under a hypoxic tumor environment (Fig. 1b and Supplementary Fig. S2a–d). Moreover, another strain of VNP_αTNF-α/mCherry_ was constructed with TNF-α nb fused to mCherry for the visualization of expressed TNF-α nb (Supplementary Fig. S1h–j). The TNF-α nb secreted by VNP had a similar particle size (75.27 ± 14.08 nm) and affinity compared with pure nanobody synthesized in our previous work^6^ (Supplementary Fig. S2e, f). VNP_αTNF-α_ induced about 40% apoptosis of B16F10 which was similar to that of VNP, while pure TNF-α nb couldn’t induce cell apoptosis, the results confirmed the antitumor activity of VNP and VNP_αTNF-α_ in vitro (Supplementary Fig. S3b, c). In addition, VNP_αTNF-α_ stimulated dendritic cells (DCs) activation and cytotoxic CD8^+^ T cell production in vitro (Fig. 1c, d). VNP_αTNF-α_ stimulated CD8^+^ T cell production immediately by activating macrophage antigen presentation (Fig. 1e, Supplementary Fig. S3d). To evaluate the neutralization ability of VNP_αTNF-α_, the supernatants of M1-type RAW264.7 were collected to measure the level of TNF-α. The result indicated that VNP_αTNF-α_ treatment significantly neutralized secreted TNF-α (sTNF-α), thus, decreasing the level of sTNF-α (Supplementary Fig. S3e).

**Fig. 1:**
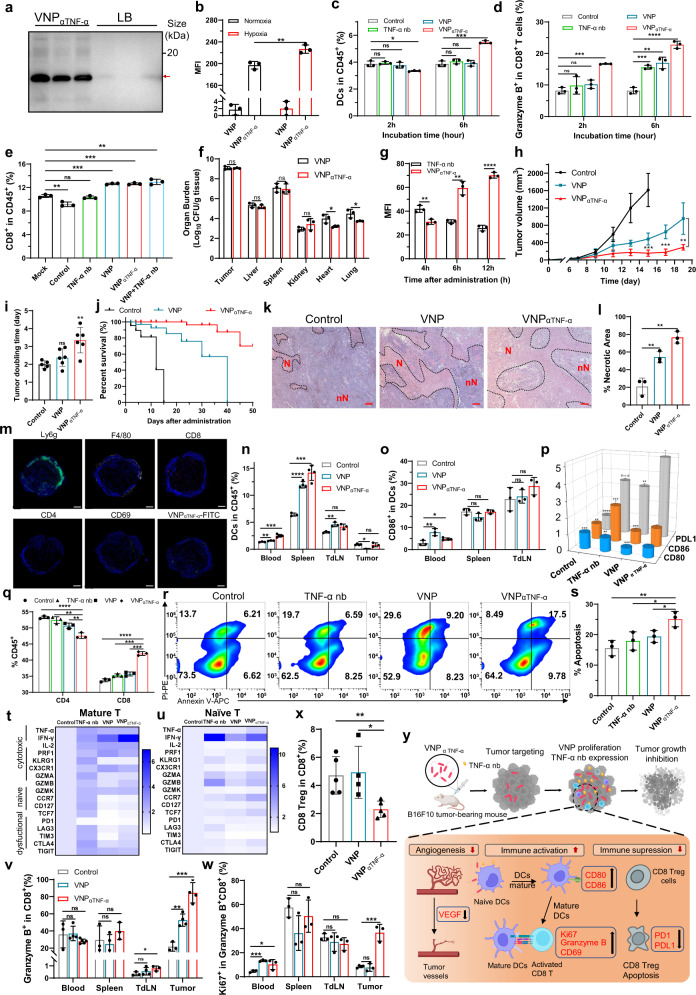
**a** The TNF-α nb expression inside bacteria or secreted into LB was detected by western blot (*n* = 3). **b** Expression of TNF-α nb-mCherry under normoxic and hypoxic (1% O_2_) conditions (*n* = 3). **c** Ratio of splenic DCs after incubated with different groups for 2 h or 6 h (*n* = 3). **d** Proportion of granzyme B^+^CD8^+^ T cells after incubated with different groups for 2 h or 6 h (*n* = 3). **e** Counted CD8^+^ T cell ratio in splenic lymphocytes after RAW264.7 cells incubation (*n* = 3). **f** The organ burden of tumor-bearing mice 5 days after injection (*n* = 3). **g** MFI analysis of representative fluorescent image of colonized VNP_αTNF-α/mCherry_ (Red; 1 × 10^8^ CFU/mouse) and TNF-α nb-mCherry (Red; 150 µg/kg) in tumors (*n* = 3). **h** Tumor growth curves of B16F10 administered with PBS, VNP, or VNP_αTNF-α_ (*n* = 6). **i** The calculated tumor doubling time of different groups (*n* = 6). **j** Overall survival of the tumor-bearing mice (*n* = 6). **k, l** H&E staining of tumors after VNP_αTNF-α_ treatments. Scale bar, 200 µm. N: necrotic region, nN: nonnecrotic region. **m** The distribution of tumor-infiltrating immune cells after VNP_αTNF-α_ administration. 1000 μm scale bars shown. (Neutrophil: Ly6g^+^; Macrophage: F4/80^+^; CD8^+^; CD4^+^; active T cells: CD69^+^; VNP_αTNF-α_: FITC^+^) **n** Quantification of DC cells in tumor and other related immune organs by flow cytometry (peripheral blood, spleen, and TdLNs) of tumor-bearing mice (*n* = 4). **o** Ratio of CD86^+^ in DCs (*n* = 4). **p** Expression of *CD80*, *CD86*, and *PDL1* in DC2.4 cells after VNP_αTNF-α_ treatment (*n* = 3). **q** Ratio of CD8^+^ and CD4^+^ T cells in the lower chambers (*n* = 3). **r, s** Apoptosis ratio of B16F10-OVA cells after VNP_αTNF-α_-stimulated splenocyte treatments (*n* = 3). **t, u** Collected unstimulated splenocytes (naïve T) and splenocytes activated by cytokine (mature T) and identified splenic T cells as naïve T cells, cytotoxic T cells, or dysfunctional T cells by RT-PCR. The bar is the relative expression level (*n* = 3). **v** Quantification of granzyme B^+^ CD8^+^ T cells in blood, spleen, TdLNs and tumor (*n* = 4). **w** Quantification of Ki67^+^granzyme B^+^ CD8^+^ T cells in blood, spleen, TdLNs and tumor (*n* = 4). **x** Flow cytometry assessment of CD8 Treg cells by CD25 and CD122 markers (*n* = 5). **y** Schematic diagram of therapeutic mechanisms of B16F10-bearing mice by VNP_αTNF-α_. Illustrations was made with BioRender. Data are shown as the mean ± SD. ****p < 0.0001, ***p < 0.001, **p < 0.01, *p < 0.05, ns no significance

To evaluate the tumor targeting ability of VNP_αTNF-α_, the organ burdens of bacteria were measured (Supplementary Fig. S4a). It was indicated that both VNP and VNP_αTNF-α_ showed tumor targeting ability as expected, which were hundreds to thousands of times higher than other tissue (Fig. 1f, Supplementary Fig. S4c). To further study the tumor residence time of TNF-α nb in tumor, we injected TNF-α nb-mCherry (150 µg/kg) and VNP_αTNF-α/mCherry_ (1 × 10^8^ CFU, the amount of secreted TNF-α nb-mCherry was equivalent to that of TNF-α nb-mCherry group) according to our data. At first, the MFI of TNF-α nb-mCherry in tumor tissue was 1.3 times higher than that of VNP_αTNF-α/mCherry_ within 4 hours. After 12 hours, pure TNF-α nb was depleted slowly, while TNF-α nb-mCherry of VNP_αTNF-α/mCherry_ increased to 2.3 times higher than pure TNF-α nb as VNP proliferated continuously (Fig. 1g, Supplementary Fig. S4b).

Next, the antitumor effect of VNP_αTNF-α_ in vivo was evaluated (Supplementary Fig. S5a). The tumor growth curve indicated that VNP_αTNF-α_ effectively inhibited melanoma progression (Fig. 1h). In addition, delayed tumor doubling time (TDT) was 2.38 days in the VNP group and 3.35 days in the VNP_αTNF-α_ group, and the TDT ratio of VNP_αTNF-α_ to PBS or VNP reached 1.67 times or 1.4 times respectively (Fig. 1i). VNP_αTNF-α_ also prolonged tumor-bearing mouse survival significantly than that of VNP (Fig. 1j). These results suggested that VNP_αTNF-α_ had an excellent therapeutic effect. Moreover, the H&E analysis of tumor section showed that more than 75% of the tumor was necrotic after VNP_αTNF-α_ treatment (Fig. 1k, l). Next, we preliminary explored the therapeutic mechanism of VNP_αTNF-α_. Firstly, it is indicated that the level of transmembrane TNF-α (tmTNF-α) in VNP_αTNF-α_ was reduced, which is lower than the baseline of the PBS group (Supplementary Fig. S6a). Since it is reported that low-dose TNF-α induces angiogenesis while high-dose TNF-α lead to thrombosis within tumor vasculature,^7^ we then assessed the distribution and gene expression of tumor vessel by CD31 and vascular endothelial growth factor (*VEGF*) staining. It is shown in Supplementary Fig. S6b, c that *VEGF* and CD31 was downregulated in the VNP_αTNF-α_ group, suggesting that VNP_αTNF-α_ inhibited tumor progression by reducing the density of tumor vessels. Therefore, VNP_αTNF-α_ induced more cell apoptosis in the tumor tissue (Supplementary Fig. S6d).

To further elucidate the therapeutic mechanisms, the distribution of tumor-infiltrating immune cells was detected. The proportion of CD8^+^ T cells and CD69^+^ cells were significantly increased, approximately 11 and 7%, while the ratio of CD4^+^ T cells were reduced in the VNP_αTNF-α_ group (Fig. 1m). In addition, the proportions of neutrophils and macrophages were significantly increased both in the VNP and VNP_αTNF-α_ group (Fig. 1m, Supplementary Fig. S7a). Next, the proportion and state of DCs in vivo were investigated. It is shown that the ratio of DCs and activated DCs (CD86^+^DCs) were significantly increased in immune organs (Fig. 1n, o). The results were consistent with in vitro experiment, where VNP_αTNF-α_ induced the upregulation expression of *CD86*, *CD80*, and *PDL1* of DC2.4 cells in vitro (Fig. 1p). As for in vivo experiment, the elevated level of *CD86* and *CD80*, and the decrease level of *PD1* and *PDL1* in the tumor mixed pool were observed (Supplementary Fig. S5b). In addition, CD11b^+^ in DCs was upregulated 1.6 times higher than that of VNP in tumor, which means DCs were activated by VNP_αTNF-α_^8^ (Supplementary Fig. S5c). Together, these results indicated that VNP_αTNF-α_ stimulated transformation form “cold” tumor with immune suppression to “hot” tumor with anti-tumor immune activation.

We further investigated whether VNP_αTNF-α_ could stimulate CD8^+^ T cell activation. Therefore, we firstly stimulated splenic T cells in vitro and cocultured them with B16F10-OVA cells as illustrated in Supplementary Fig. S5d, the cells in lower chambers were collected and tumor cell-recruited CD8^+^ T cells and B16F10-OVA cells apoptosis were analyzed. The results revealed that the highest proportion of CD45^+^cells in the lower chamber was T cells, which was approximately 90%, and the proportion of CD8^+^ T cells increased from 32 to 40%, in contrast, CD4^+^ T cells decreased after VNP_αTNF-α_ incubation (Fig. 1q), which indicated that VNP_αTNF-α_ stimulated CD8^+^ T cell chemotaxis and activation. As a result, significant tumor apoptosis was induced from 15 to 25% by VNP_αTNF-α_-stimulated splenic T cells (Fig. 1r, s). Further detection of relative expression of markers by RT-PCR showed that VNP_αTNF-α_ induced CD8^+^ T cell polarization into cytotoxic T cells, according to the upregulated *TNF-α, IFN-γ, IL-2, perforin*, and *granzyme B* as well as downregulated markers of exhausted cells, such as *PD1* and *TIM3*. More importantly, in vivo experiments showed that splenic CD8^+^ T cells matured after stimulation because the markers of naïve T cells were downregulated (*CCR7* and *TCF7*) (Fig. 1t, u). In addition, the percentage of granzyme-B^+^ CD8^+^ T cells of VNP_αTNF-α_ group was increased in immune organs, particularly in tumor, which was four times higher than the control group (Fig. 1v). And VNP_αTNF-α_ stimulated more Ki67^+^ cytotoxic CD8^+^ T cell, which was five times higher than the control group (Fig. 1w). These results indicated that VNP_αTNF-α_ mobilized the systemic immune response. Furthermore, it is noteworthy that VNP_αTNF-α_ reduced CD8^+^ T cell death approximately two-fold (Supplementary Fig. S5f). Notably, the same results were previously reported that anti-TNF-α inhibitor lessened CD8^+^ T cell death in vivo.^9^ Meanwhile, CD8 Tregs were reduced in the tumor draining lymph nodes (TdLNs) and tumor after VNP_αTNF-α_ administration (Fig. 1x, Supplementary Fig. S5g, i). As expected, the percentage of Annexin V-positive CD8 Tregs was increased approximately twice after VNP_αTNF-α_ administration (Supplementary Fig. S5h), which means that the tumor immunosuppression was alleviated.

Based on our strategy, TNF-α nb could be delivered into tumor tissue by VNP safely and efficiently, and this system exerted robust antitumor effects with controllable TNF-α nb secretion in melanoma with only one dosage, which could also avoid the side effects and high costs of TNF-α inhibitors. Moreover, VNP_αTNF-α_ promoted antitumor immune responses in a melanoma tumor microenvironment by mobilizing tumor immune response as follows, (1) VNP_αTNF-α_ reduced tumor angiogenesis. (2) VNP_αTNF-α_ stimulated DCs maturation manifesting as elevated CD86. DCs activated CD8^+^ T cells by antigen-presentation and induced CD8^+^ T cells to upregulate Granzyme-B and Ki67, stimulated cytotoxic CD8^+^ T cell induced tumor apoptosis. (3) CD8 Treg reduced after administration of VNP_αTNF-α_. (4) VNP_αTNF-α_ directly induced tumor apoptosis in vivo and in vitro (Fig. 1y). In addition, VNP_αTNF-α_ causes acceptable splenomegaly but has better biosafety than VNP (Supplementary Fig. S8a–f).

## Supplementary information

supporting information (clean)-7742R1

## Data Availability

All data generated or analyzed during this study are included either in this article or in the supplementary information files.
