# Supplementary material for: A bacteria-based system expressing anti-TNF-α nanobody for enhanced cancer immunotherapy
Source: Signal Transduct Target Ther. 2023 Apr 19;8:134. doi: 10.1038/s41392-023-01364-0 (PMC10113364; doi:10.1038/s41392-023-01364-0)
Supplement: Supplementary file 1 — supporting information (clean)-7742R1 [file 41392_2023_1364_MOESM1_ESM.docx]

Supplementary Materials for

A bacteria-based system expressing anti-TNF-α nanobody for enhanced cancer immunotherapy

Lina Liu^1^, Xing Liu^1^, Wenjie Xin^1^, Lulu Zhou^3^, Baolian Huang^3^, Chao Han^1^, Zhiting Cao^3*^, Zichun Hua^1, 2, 3*^

*Correspondence authors to: **Zichun Hua** (E-mail: zchua@nju.edu.cn) and **Zhiting Cao** (E-mail: caozt@cpu.edu.cn)

^1^ The State Key Laboratory of Pharmaceutical Biotechnology, School of Life Sciences; Nanjing University, Nanjing, 210023, Jiangsu, China.

^2^ Changzhou High-Tech Research Institute of Nanjing University and Jiangsu TargetPharma Laboratories Inc.; Changzhou, 213164, Jiangsu, China.

^3^ School of Biopharmacy, China Pharmaceutical University; Nanjing, 210023, Jiangsu, China

**This PDF file includes:**

Materials and Methods

Figures. S1 to S8

Tables S1

Tables S2

**Materials and Methods**

Materials.

The AuNPs were preparate following previous reported ^10^. AuNPs incubated with TNF-α (1 µg/ml) at 25 ℃ in 30 minutes. The sample was concentrated by ultrafiltration to 500 µl (Amicon Ultra-15, 3 kDa, millipore). AuNPs-TNF-α mixed with VNP_TNF-α_ culture medium at 25 ℃ in 15 minutes for cryo-electron microscope experiments (Cryo-TEM).

Mouse Model.

Animal experiments were performed as per the appropriate ethical guidelines of the Nanjing University Animal Care and Use Committee. Female C57BL/6J, 6 to 8 weeks old, were purchased from Huachuang Sino Company (Nanjing, China) and housed under constant pathogen-free conditions. To establish a melanoma xenograft model, C57BL/6J mice was subcutaneously (*s.c.*) injected with 5×10^5^ B16F10 cells. After 7 days, 1×10^6^ CFU VNP were injected intraperitoneally (*i.p.*) into tumor-bearing mice. Then 5 days after the VNP injection, the mice were executed and collected tumor, spleen, tumor-draining lymph nodes (TdLN), and peripheral blood. The mouse condition and body weight were monitored closely. Tumor diameter was measured with Vernier calipers.

Whole peripheral blood samples were placed at room temperature for 2 hours, and then at 2-8℃ at 3000 rpm, for 15min, and the supernatant was collected to detect aspartate aminotransferase (AST), alanine aminotransferase (ALT), UREA, and CREA. The experiments were completed by Wuhan servicebio technology company (Wuhan, China).

Cell Culture and Splenocytes Stimulation.

B16F10 mouse melanoma cells and RAW 264.7 mouse macrophage cells were archived in our laboratory. B16F10 cells were cultured in RPMI-1640 medium (BBI,China) containing 10% fetal bovine serum (HyClone, USA). RAW 264.7 cells were cultured in Dulbecco’s modified Eagle medium (DMEM) (BBI,China) containing 15% FBS (Gibco,USA). All the cell lines were cultured at 37 ℃ in an incubator with 5% CO2.

Splenocytes were collected from 6-8 weeks old female C57BL/6J spleen, and single cell were obtained by homogenization with 1 ml PBS. Red Blood Cell Lysis Buffer (Beyotime, Nanjing) was used to lyse red blood cells. To harvest mature CD8^+^T cells, spleen single cells were cultured in RPMI-1640 medium (BBI, China) containing 20% FBS (Gibico, USA), and anti-CD3(5 μg/ml, PeproTech, US), anti-CD28 (5 μg/mL, PeproTech, US), IL-2 (10 ng/ml, PeproTech,US) and OVA_257-264_ (500 ng/ml, GenScript, China) were added to the indicated condition, constantly stimulated splenocytes in 2 days.

Bacterial strains and plasmid.

VNP20009 (VNP) and J23100 initiate VNP20009 expressing TNF-α nb (VNP_αTNF-α_), and VNP_αTNF-α/mCherry_ (VNP expressing TNF-α nb-mcherry ) cultured in Luria Bertani (LB) broth or on LB agar plates, at 37℃. The bacterial growth curve constantly detected the OD_600_ for 24 h by a microplate reader. The expression plasmid of VNP_αTNF-α_ was constructed by using the ClonExpress II/MultiS One Step Cloning Kit (C112/C113, Vazyme), and the plasmid backbone is based on pET32a modification. And a strong constitutive promoter and anti-plasmid loss element were inserted into the expression plasmid for continuous TNF-α nb expression *in vivo*, and pelB, a signal peptide, and a flag tag were fused with TNF-α nb to lead the expressed TNF-α nb to the periplasmic space (PPS) in a hypoxic environment.

When OD_600_ reached 0.6-0.8, used sonication lysis bacterial to measure the expression of TNF-α nb in bacteria. TCA-acetone precipitation was used to extract protein in LB 8 hours after culture VNP_αTNF-α_.

Real-Time PCR.

Total RNA was isolated using TRIzol Reagent (Vazyme, China). The cDNA templates were reverse transcribed from 1 µg RNA with a TUYOBO cDNA Synthesis kit (TUYOBO, Japan) according to the manufacturer’s instructions. Relative mRNA levels were detected using One Step RT-PCR SYBR Green Kit (Vazyme, China) following manufacturer’s instructions. The primers were synthesized by Genscript (Nanjing, China) and are listed in **Additional file 1, Table 1**.

Western Blot and Sliver staining.

Bacteria were cultured with LB at 37℃, until growth to OD_600_.=0.6-0.8. The bacteria were harvested using centrifugation and disrupted using sonication. All protein samples were subjected to 12% SDS-polyacrylamide gel electrophoresis and transferred to PVDF membranes. The western blot antibody used was as follows: ANTI-FLAG®M2 antibody (B3111-1MG) from Sigma (USA).

Silver staining was performed with the Fast Silver Stain Kit (Beyotime, Nanjing) following the manufacturer’s recommendations.

Flow Cytometry.

Five days after VNP injection, tumor-bearing mouse tissues were collected. peripheral blood lymphocytes were obtained from peripheral blood. spleen and TdLN were homogenized with 1 ml PBS to obtain single-cell suspensions. Tumors were shredded and then digested with mixed medium (1 mg/ml Collagenase I, 1 mg/ml Collagenase IV, 200 µg /ml DNase I) at 37℃ for 40min. All tissues were lysed with red blood cell lysis buffer (Beyotime, Nanjing) lysis red blood cells, and then the cell suspensions were passed through a 200-mesh filter.

The single-cell suspensions were incubated in 1% BSA for 15 minutes at 4℃ and stained with the following antibodies for 30 minutes at 4℃ (detailed antibody information is provided in **Additional file 1, Table 2**. The stained cells were analyzed using flow cytometer (BD@ FACS Canto II systems). The results were analyzed using FlowJo VX software.

Cell apoptosis detection.

The apoptosis levels of B16F10 were determined using a kit developed by our laboratory. Then, 1 µl Annexin V protein (1 mg/ml) and 1 µl propidium (PI, 1 mg/ml) were added to 1×10^6^ cells and incubated in binding buffer (10 mM HEPS, 140 mM NaCl, 2.5 mM CaCl_2_) for 30 minutes at 4℃. The stained cells were analyzed using a flow cytometer (NovoCyte Flow Cytometer (ACEA@)). The results were analyzed using FlowJo VX software.

ELISA.

RAW264.7 were stimulated by 100ng/ml LPS for 24 hours, and nanobody (10 μg/ml) and VNP_αTNF-α_ (2×10^7^ CFU) were incubated with M1-type RAW for 6 hours. Next, the supernatants were collected to measure the level of TNF-α. According to ELISA was performed according to TNF-α ELISA kit (FMS-ELM028, Fcmacs Biotech Co, China) instructions.

HE and Immunofluorescence.

B10F10-bearing mice were sacrificed 5 days after administration. And the tissues, such as tumor, spleen, liver, kidney, lung, and heart, were collected to made paraffin sections. HE stains were conducted on section. Immunofluorescence was carried out as described previously. The experiments were completed by Wuhan servicebio technology company (Wuhan, China).

Statistical Analysis.

Results are expressed as the mean ± SD as specified. Mean differences were compared using *t*-test or one-way ANOVA. A value of *p*<0.05 was regarded as statistically significant. Data were analyzed with GraphPad Prism 8.3 software. ( **** *P* < 0.0001, *** *P* < 0.001, ** *P* < 0.01, * *P* < 0.05.)

**Supplementary Figures**


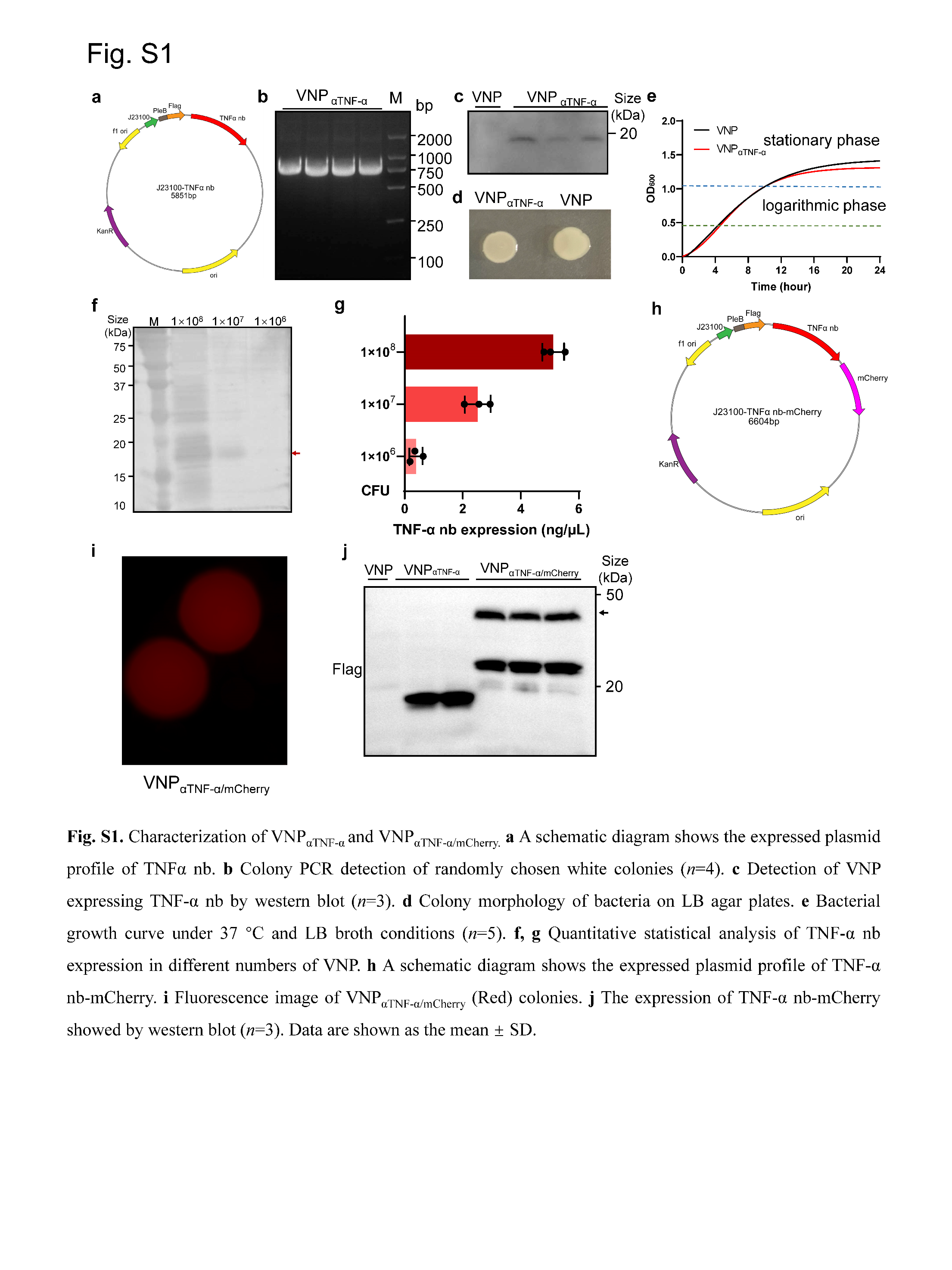


**Fig. S1.** Characterization of VNP_αTNF-α_ and VNP_αTNF-α/mCherry._ **a** A schematic diagram shows the expressed plasmid profile of TNFα nb. **b** Colony PCR detection of randomly chosen white colonies (*n*=4). **c** Detection of VNP expressing TNF-α nb by western blot (*n*=3). **d** Colony morphology of bacteria on LB agar plates. **e** Bacterial growth curve under 37 °C and LB broth conditions (*n*=5). **f, g** Quantitative statistical analysis of TNF-α nb expression in different numbers of VNP. **h** A schematic diagram shows the expressed plasmid profile of TNF-α nb-mCherry. **i** Fluorescence image of VNP_αTNF-α/mCherry_ (Red) colonies. **j** The expression of TNF-α nb-mCherry showed by western blot (*n*=3). Data are shown as the mean ± SD.


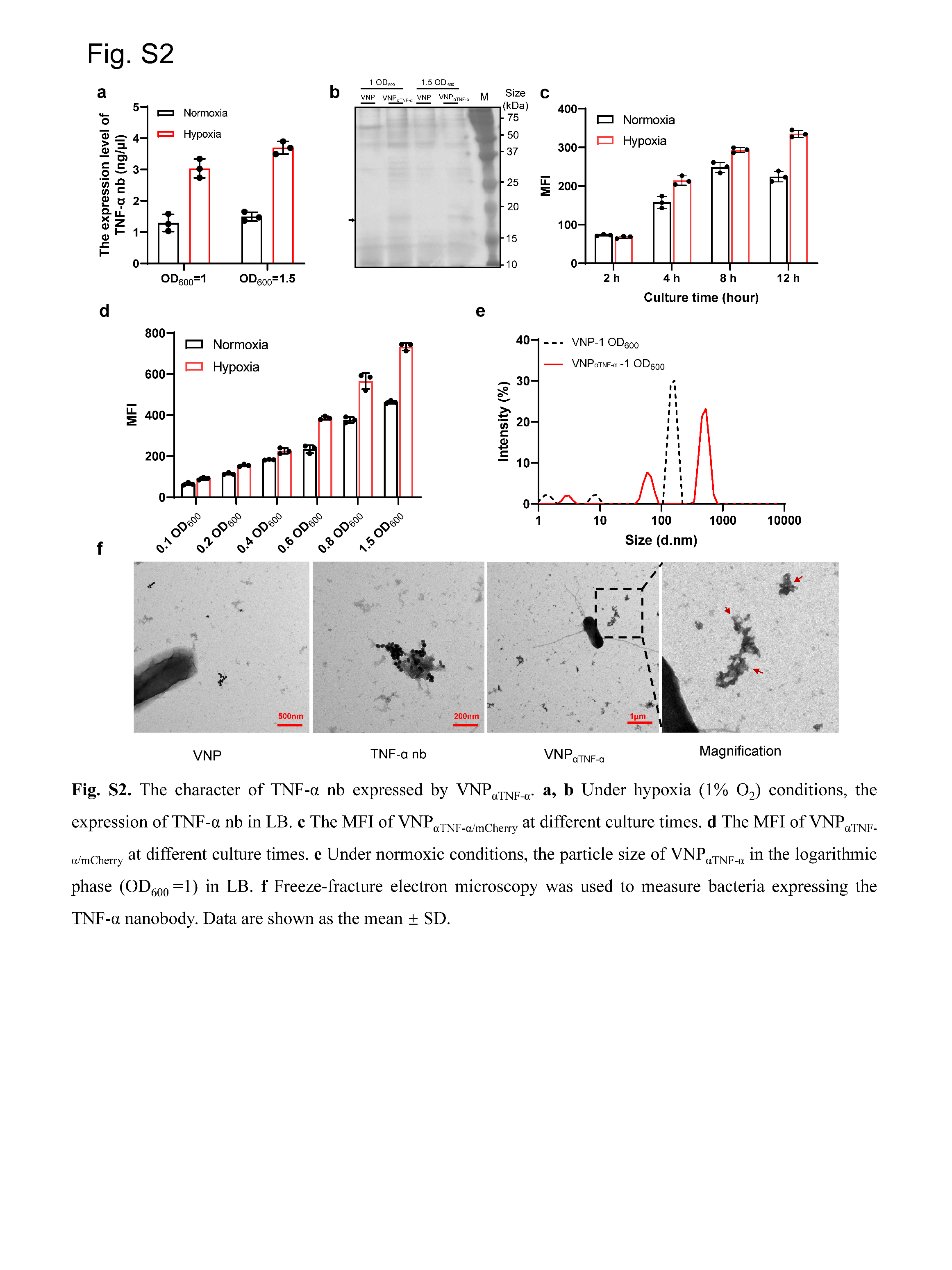


**Fig. S2.** The character of TNF-α nb expressed by VNP_αTNF-α_. **a, b** Under hypoxia (1% O_2_) conditions, the expression of TNF-α nb in LB (*n*=3). **c** The MFI of VNP_αTNF-α/mCherry_ at different culture times (*n*=3). **d** The MFI of VNP_αTNF-α/mCherry_ at different culture times (*n*=3). **e** Under normoxic conditions, the particle size of VNP_αTNF-α_ in the logarithmic phase (OD_600_ =1) in LB. **f** Freeze-fracture electron microscopy was used to measure bacteria expressing the TNF-α nanobody. Data are shown as the mean ± SD.


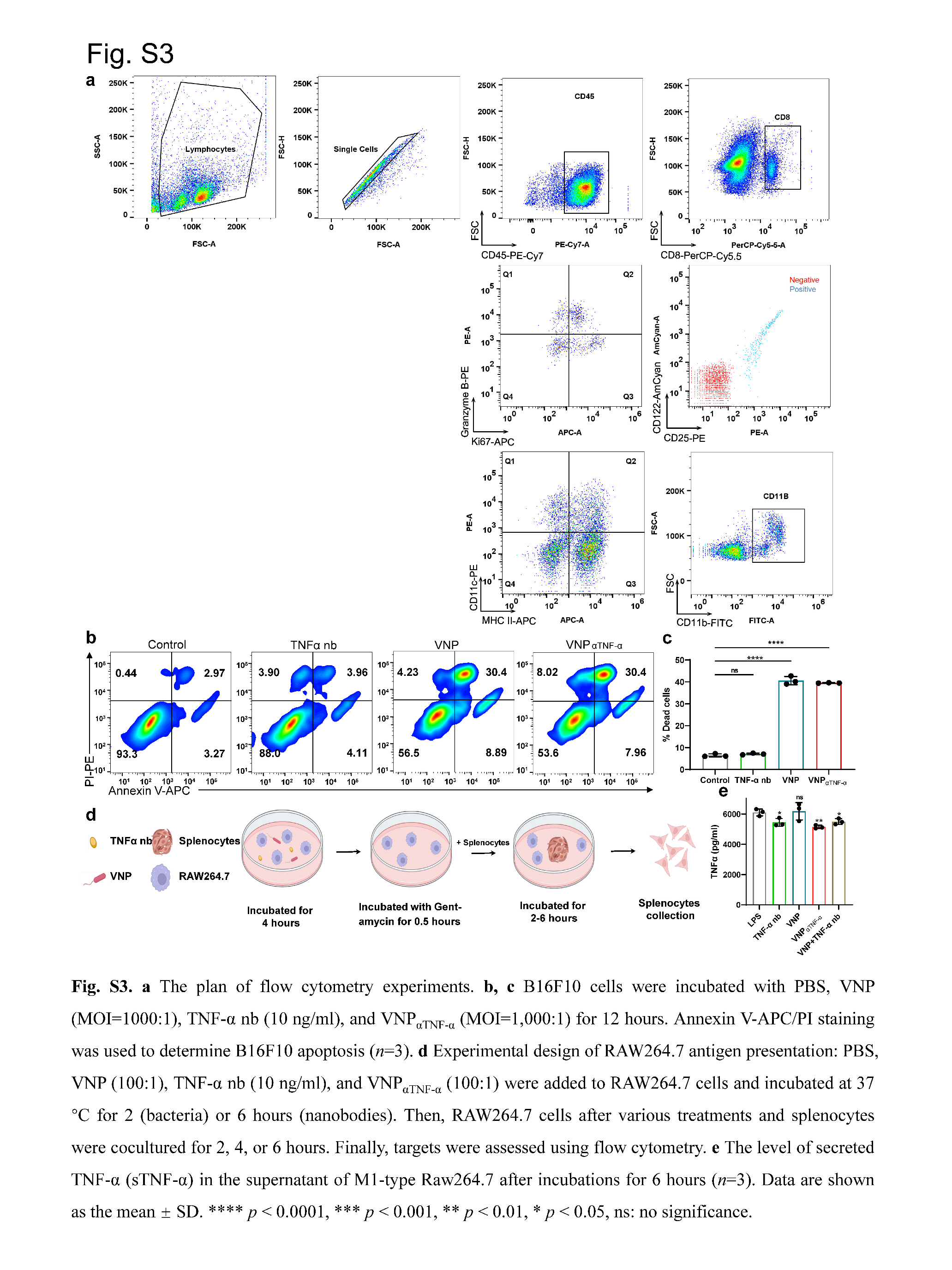


**Fig. S3. a** The plan of flow cytometry experiments. **b, c** B16F10 cells were incubated with PBS, VNP (MOI=1000:1), TNF-α nb (10 ng/ml), and VNP_αTNF-α_ (MOI=1,000:1) for 12 hours. Annexin V-APC/PI staining was used to determine B16F10 apoptosis (*n*=3). **d** Experimental design of RAW264.7 antigen presentation: PBS, VNP (100:1), TNF-α nb (10 ng/ml), and VNP_αTNF-α_ (100:1) were added to RAW264.7 cells and incubated at 37 °C for 2 (bacteria) or 6 hours (nanobodies). Then, RAW264.7 cells after various treatments and splenocytes were cocultured for 2, 4, or 6 hours. Finally, targets were assessed using flow cytometry. **e** The level of secreted TNF-α (sTNF-α) in the supernatant of M1-type Raw264.7 after incubations for 6 hours (*n*=3). Data are shown as the mean ± SD. **** *p* < 0.0001, *** *p* < 0.001, ** *p* < 0.01, * *p* < 0.05, ns: no significance.


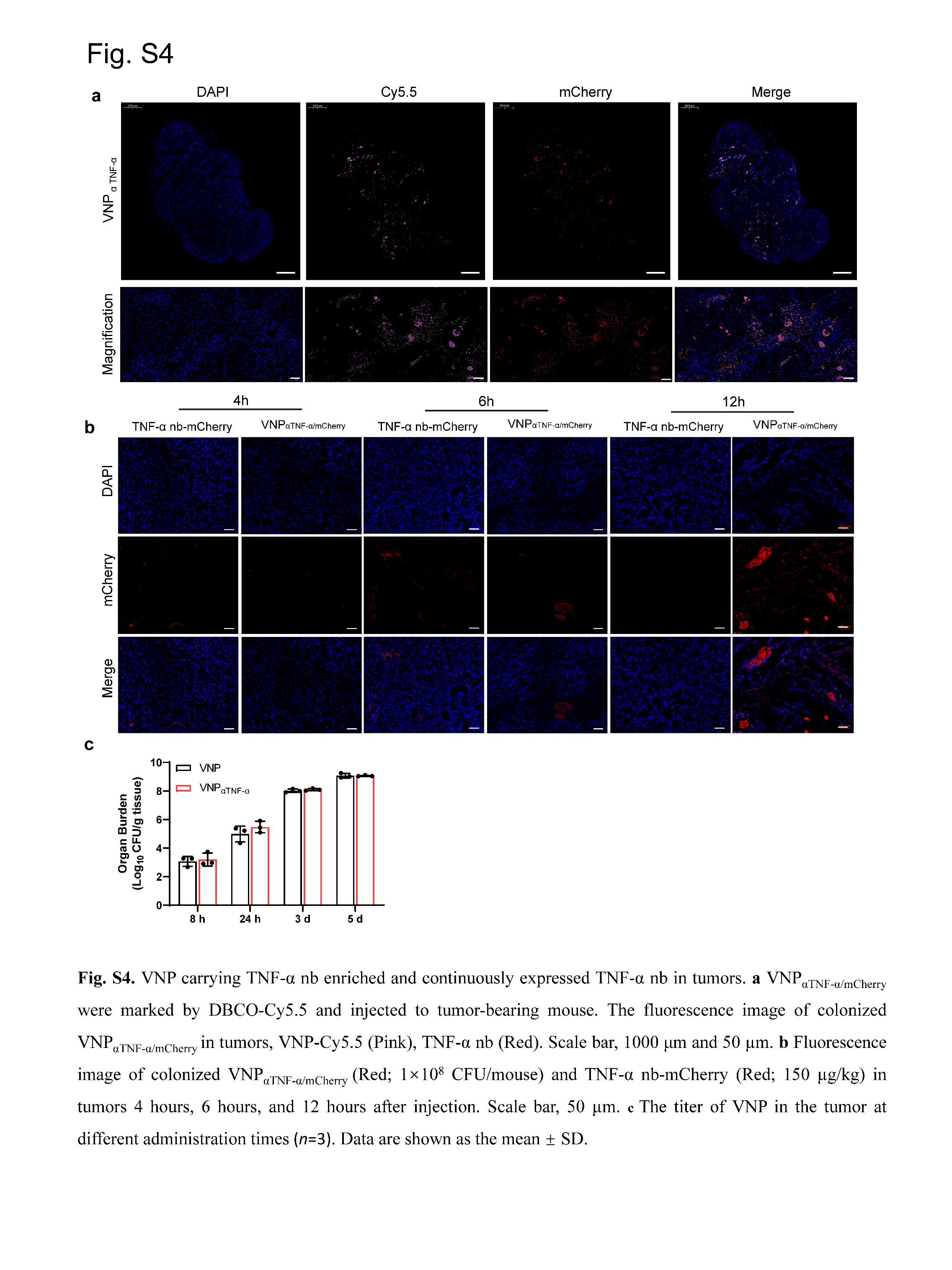


**Fig. S4.** VNP carrying TNF-α nb enriched and continuously expressed TNF-α nb in tumors. **a** VNP_αTNF-α/mCherry_ were marked by DBCO-Cy5.5 and injected to tumor-bearing mouse. The fluorescence image of colonized VNP_αTNF-α/mCherry_ in tumors, VNP-Cy5.5 (Pink), TNF-α nb (Red). Scale bar, 1000 μm and 50 µm. **b** Fluorescence image of colonized VNP_αTNF-α/mCherry_ (Red; 1×10^8^ CFU/mouse) and TNF-α nb-mCherry (Red; 150 µg/kg) in tumors 4 hours, 6 hours, and 12 hours after injection. Scale bar, 50 µm. **c** The titer of VNP in the tumor at different administration times (*n*=3). Data are shown as the mean ± SD.


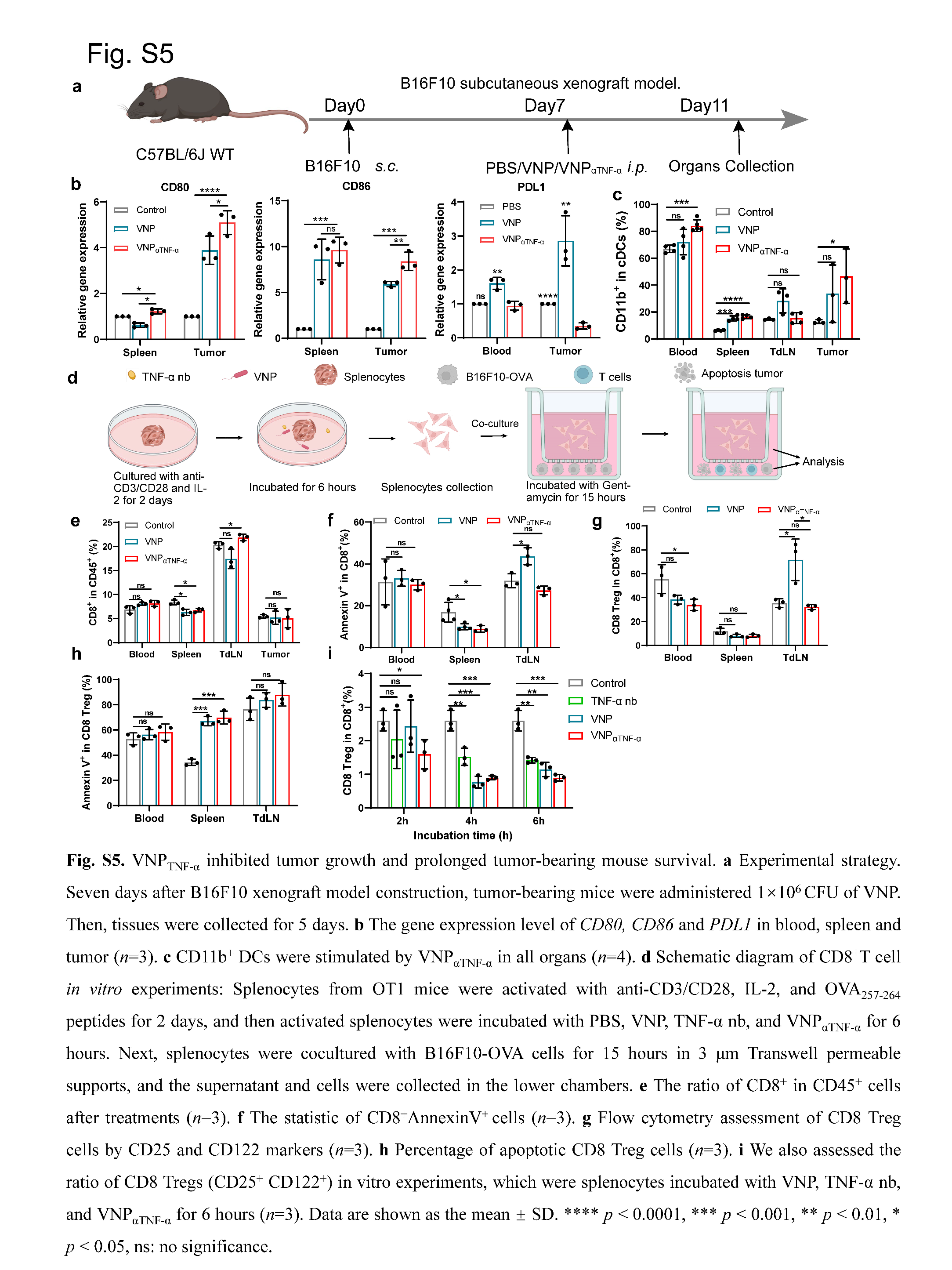


**Fig. S5.** VNP_αTNF-α_ inhibited tumor growth and prolonged tumor-bearing mouse survival. **a** Experimental strategy. Seven days after B16F10 xenograft model construction, tumor-bearing mice were administered 1×10^6^ CFU of VNP. Then, tissues were collected for 5 days. **b** The gene expression level of *CD80,* *CD86* and *PDL1* in blood, spleen and tumor (*n*=3). **c** CD11b^+^ DCs were stimulated by VNP_αTNF-α_ in all organs (*n*=4). **d** Schematic diagram of CD8^+^T cell *in vitro* experiments: Splenocytes from OT1 mice were activated with anti-CD3/CD28, IL-2, and OVA_257-264_ peptides for 2 days, and then activated splenocytes were incubated with PBS, VNP, TNF-α nb, and VNP_αTNF-α_ for 6 hours. Next, splenocytes were cocultured with B16F10-OVA cells for 15 hours in 3 μm Transwell permeable supports, and the supernatant and cells were collected in the lower chambers. **e** The ratio of CD8^+^ in CD45^+^ cells after treatments (*n*=3). **f** The statistic of CD8^+^AnnexinV^+^ cells (*n*=3). **g** Flow cytometry assessment of CD8 Treg cells by CD25 and CD122 markers (*n*=3). **h** Percentage of apoptotic CD8 Treg cells (*n*=3). **i** We also assessed the ratio of CD8 Tregs (CD25^+^ CD122^+^) in vitro experiments, which were splenocytes incubated with VNP, TNF-α nb, and VNP_αTNF-α_ for 6 hours (*n*=3). Data are shown as the mean ± SD. **** *p* < 0.0001, *** *p* < 0.001, ** *p* < 0.01, * *p* < 0.05, ns: no significance.


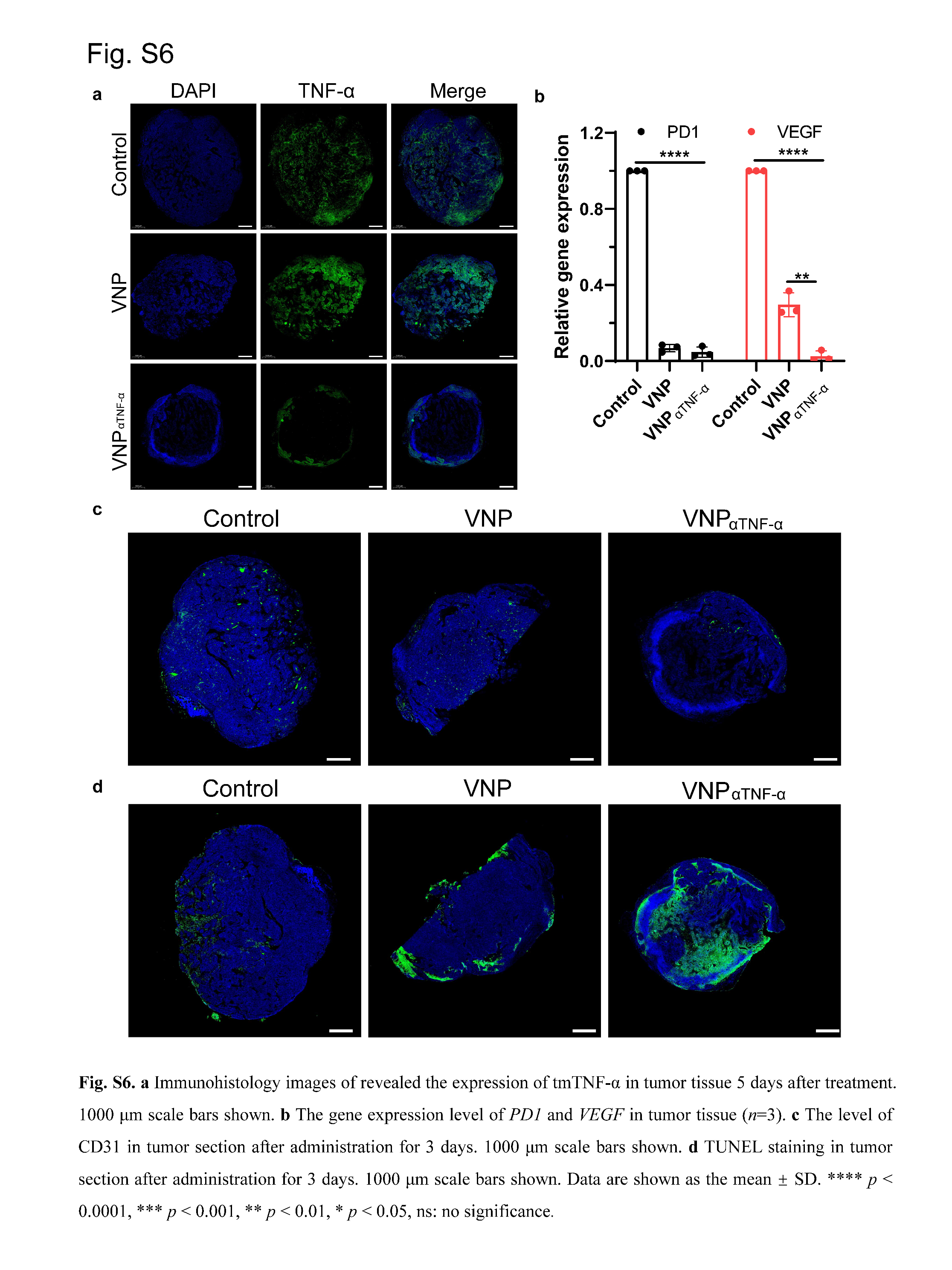


**Fig. S6. a** Immunohistology images of revealed the expression of tmTNF-α in tumor tissue 5 days after treatment. 1000 μm scale bars shown. **b** The gene expression level of *PD1* and *VEGF* in tumor tissue (*n*=3). **c** The level of CD31 in tumor section after administration for 3 days. 1000 μm scale bars shown. **d** TUNEL staining in tumor section after administration for 3 days. 1000 μm scale bars shown. Data are shown as the mean ± SD. **** *p* < 0.0001, *** *p* < 0.001, ** *p* < 0.01, * *p* < 0.05, ns: no significance.


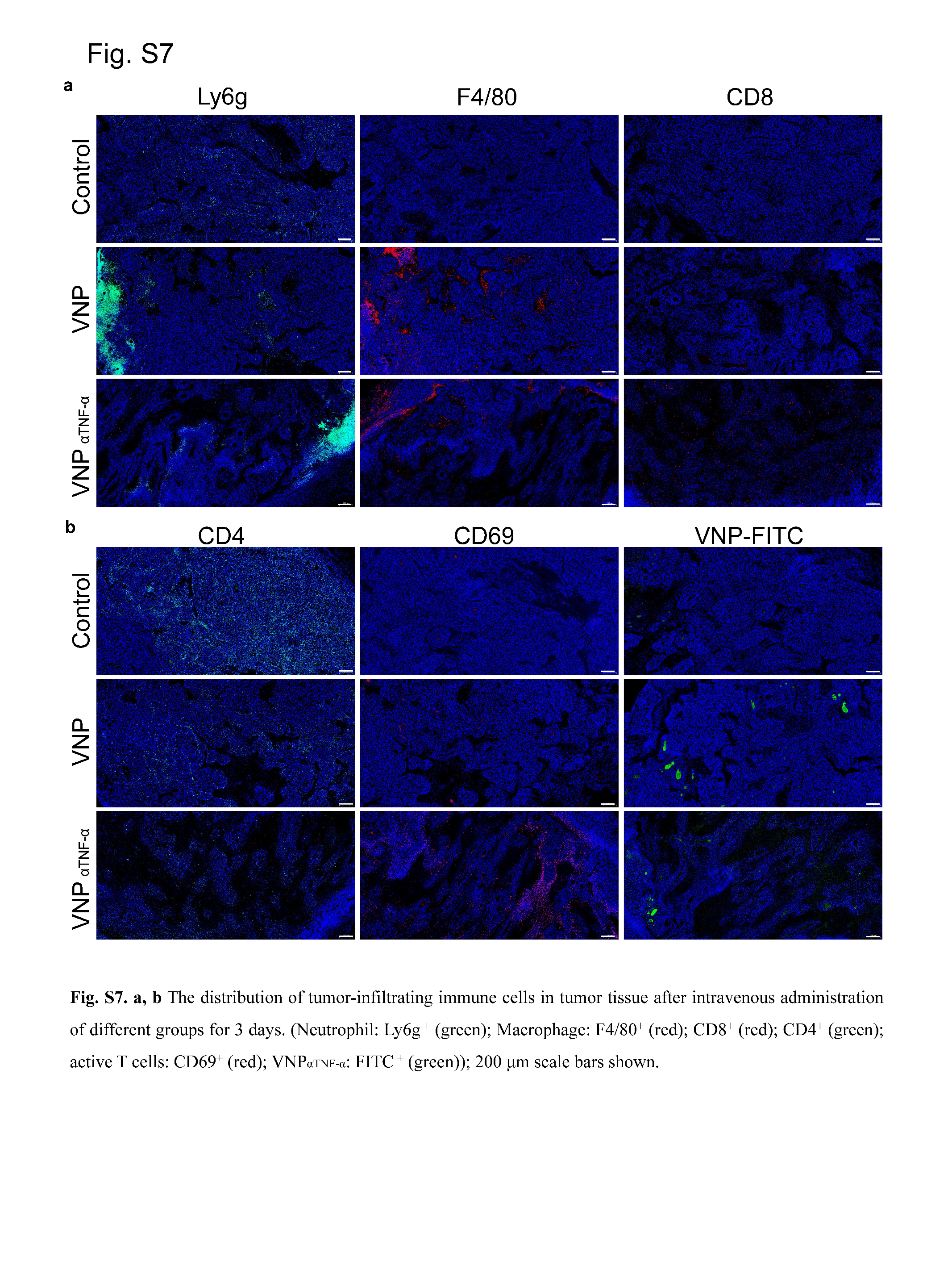


**Fig. S7. a, b** The distribution of tumor-infiltrating immune cells in tumor tissue after intravenous administration of different groups for 3 days. (Neutrophil: Ly6g ^+^ (green); Macrophage: F4/80^+^ (red); CD8^+^ (red); CD4^+^ (green); active T cells: CD69^+^ (red); VNP_αTNF-α:_ FITC ^+^ (green)); 200 μm scale bars shown. Data are shown as the mean ± SD.


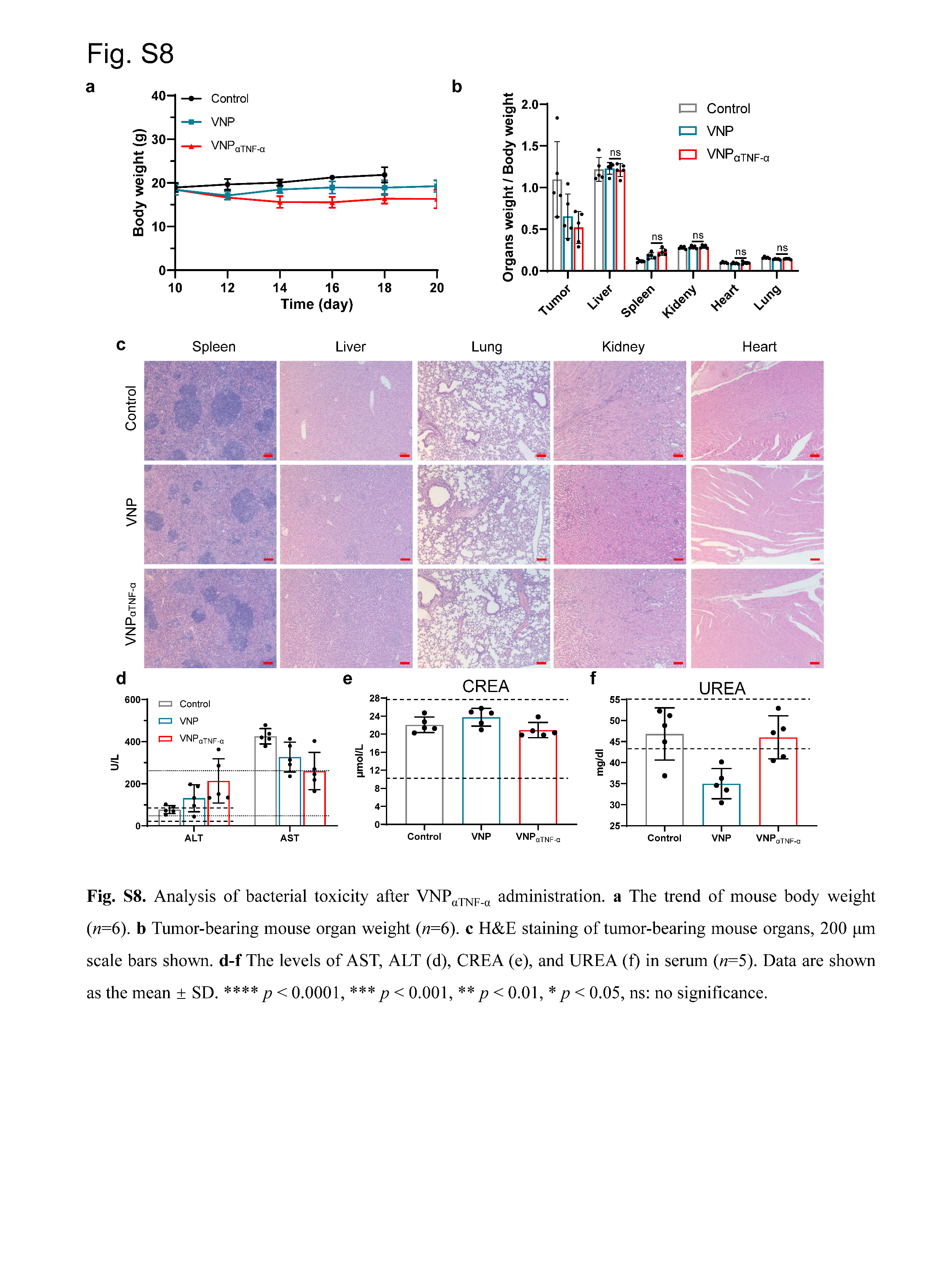


**Fig. S8.** Analysis of bacterial toxicity after VNP_αTNF-α_ administration. **a** The trend of mouse body weight (*n*=6). **b** Tumor-bearing mouse organ weight (*n*=6). **c** H&E staining of tumor-bearing mouse organs, 200 μm scale bars shown. **d-f** The levels of AST, ALT (d), CREA (e), and UREA (f) in serum (*n*=5). Data are shown as the mean ± SD. **** *p* < 0.0001, *** *p* < 0.001, ** *p* < 0.01, * *p* < 0.05, ns: no significance.

**Table S1. The primer sequence of RT-PCR.**

| **Primer** | **Forward (5'-3')** | **Reverse（5'-3'）** |
| --- | --- | --- |
| 18s | GTAACCCGTTGAACCCCATT | CCATCCAATCGGTAGTAGCG |
| IFN-γ | GCCACGGCACAGTCATTGA | TGCTGATGGCCTGATTGTCTT |
| TNFα | ACCACGCTCTTCTGTCTACT | AGGAGGTTGACTTTCTCCTG |
| IL-2 | GTGCTCCTTGTCAACAGCG | GGGGAGTTTCAGGTTCCTGTA |
| TNFR1 | AGAACCAGTTCCAACGCTACC | TCTGAGTCTCCTTACAGGGGAT |
| TNFR2 | CAGGTTGTCTTGACACCCTAC | GCACAGCACATCTGAGCCT |
| GZMB | CCACTCTCGACCCTACATGG | GGCCCCCAAAGTGACATTTATT |
| GZMA | TGCTGCCCACTGTAACGTG | GGTAGGTGAAGGATAGCCACAT |
| GZMK | TGGCTGGCGTTTATATGTCTTC | GCTGCGGTACTGGATGGAC |
| CX3CR1 | GAGTATGACGATTCTGCTGAGG | CAGACCGAACGTGAAGACGAG |
| KLRG1 | GGCTCACATCTCCTTACATTTCC | CAAGCCGATCCAGTAAAAGTCC |
| PRF1 | CTGCCACTCGGTCAGAATG | CGGAGGGTAGTCACATCCAT |
| CCR7 | TGTACGAGTCGGTGTGCTTC | GGTAGGTATCCGTCATGGTCTTG |
| CD127 | GCGGACGATCACTCCTTCTG | AGCCCCACATATTTGAAATTCCA |
| TCF7 | AGCTTTCTCCACTCTACGAACA | AATCCAGAGAGATCGGGGGTC |
| LAG3 | CTGGGACTGCTTTGGGAAG | GGTTGATGTTGCCAGATAACCC |
| TIGIT | GAATGGAACCTGAGGAGTCTCT | AGCAATGAAGCTCTCTAGGCT |
| PD1 | ACCCTGGTCATTCACTTGGG | CATTTGCTCCCTCTGACACTG |
| TIM3 | ACTGGTGACCCTCCATAATAACA | GCAGTTCTGATCGTTTCTCCA |
| CTLA4 | TTTTGTAGCCCTGCTCACTCT | CTGAAGGTTGGGTCACCTGTA |
| CD80 | ACCCCCAACATAACTGAGTCT | TTCCAACCAAGAGAAGCGAGG |
| CD86 | CTGGACTCTACGACTTCACAATG | AGTTGGCGATCACTGACAGTT |
| PDL1 | GCTCCAAAGGACTTGTACGTG | TGATCTGAAGGGCAGCATTTC |

**Table S2. The information of FACs antibody.**

| **Antibodies** | **Article No.** | **Company** |
| --- | --- | --- |
| Ms CD45-PE Cy7 | 552848 | BD Pharmingen™ |
| Ms CD8a-PerCP Cy5.5 | 551162 | BD Pharmingen™ |
| Ms CD11c-PE | 553802 | BD Pharmingen™ |
| Ms CD274 BV421 | 564716 | BD Pharmingen™ |
| Ms CD11b- FITC | 557396 | BD Pharmingen™ |
| Ms CD122-BV510 | 740118 | BD Pharmingen™ |
| MHC Class II-APC | 17-5321-81 | eBioscience™ |
| Ki67-AF647 | 17-5698-82 | eBioscience™ |
| Granzyme B-PE | 12-8898-82 | eBioscience™ |
| CD86-FITC | 105005 | Biolegend™ |

**Reference**

10 Ma, Y. *et al.* Near-infrared II phototherapy induces deep tissue immunogenic cell death and potentiates cancer immunotherapy. *ACS Nano* **13**, 11967–11980 (2019).
